# Antiresorptive Drugs and the Risk of Femoral Shaft Fracture in Men and Women With Osteoporosis: A Cohort Study Using the National Database of Health Insurance Claims of Japan

**DOI:** 10.2188/jea.JE20220099

**Published:** 2023-12-05

**Authors:** Takumi Imai, Takayuki Hosoi, Hiroshi Hagino, Takanori Yamamoto, Tatsuhiko Kuroda, Hiroshi Watanabe, Shiro Tanaka

**Affiliations:** 1 Department of Medical Statistics, Graduate School of Medicine, Osaka Metropolitan University, Osaka, Japan; 2 Kenkoin Clinic, Tokyo, Japan; 3 School of Health Science, Tottori University Faculty of Medicine, Tottori, Japan; 4 Medical Affairs Capabilities, Japan Drug Development and Medical Affairs, Eli Lilly Japan K.K., Kobe, Japan; 5 Public Health Research Foundation, Tokyo, Japan; 6 Department of Clinical Research and Development, National Center for Geriatrics and Gerontology, Aichi, Japan; 7 Department of Clinical Biostatistics, Graduate School of Medicine, Kyoto University, Kyoto, Japan

**Keywords:** claims database, femoral shaft fracture, osteoporosis, pharmacoepidemiology, Japanese population

## Abstract

**Background:**

This cohort study aimed to estimate incidence rates of femoral shaft fracture in patients who were treated with antiresorptive drugs.

**Methods:**

We used data from the National Database of Health Insurance Claims of Japan from April 2009 and October 2016. All patients with new use of an antiresorptive drug, prescription-free period of ≥3 months, and no prior femoral fractures were included. Femoral shaft fractures were identified using a validated definition based on International Classification of Diseases, 10^th^ revision (ICD-10) codes. Incidence rate ratios were estimated using Poisson regression, with adjustment for sex, age, and the Charlson Comorbidity Index.

**Results:**

We identified 7,958,655 patients (women: 88.4%; age ≥75 years: 51.2%). Femoral shaft fractures were identified in 22,604 patients. Incidence rates per 100,000 person-years were 74.8 for women, 30.1 for men, 30.1 for patients aged ≤64 years, 47.7 for patients aged 65–74 years, and 99.0 for patients aged ≥75 years. Adjusted incidence rate ratios in patients taking versus not taking each type of antiresorptive drug were 1.00 (95% confidence interval [CI], 0.98–1.03) for bisphosphonates, 0.46 (95% CI, 0.44–0.48) for selective estrogen receptor modulators, 0.24 (95% CI, 0.18–0.32) for estrogens, 0.75 (95% CI, 0.71–0.79) for calcitonins, and 0.93 (95% CI, 0.84–1.03) for denosumab. The adjusted incidence rate ratio for alendronate was 1.18 (95% CI, 1.14–1.22).

**Conclusion:**

The incidence rates of femoral shaft fracture varied across patients treated with different antiresorptive drugs. Further research on a specific antiresorptive drug can increase understanding of the risk of femoral shaft fracture.

## INTRODUCTION

Bisphosphonates, a standard pharmacological treatment for osteoporosis, increase bone mineral density and reduce the risk of incident fracture via inhibition of osteoclast-mediated bone resorption.^1^ The first case report of an unusual fragility fracture, an atypical femoral fracture (AFF), involved a patient with osteoporosis taking a bisphosphonate in 2006.^2^ Thereafter, several reports on the relationship between AFFs and long-term bisphosphonate therapy were published.^3^^–^^6^ It is possible that other antiresorptive drugs, such as denosumab,^7^^,^^8^ increase the risk of AFF. In general, AFFs have been defined as femoral shaft or subtrochanteric femoral fractures after minimal or no trauma.^9^

Previous cohort studies have mainly focused on the associations between bisphosphonates and AFF, although AFF may be caused by other antiresorptive drugs.^10^^,^^11^ Among patients in the Healthy Bones Program at Kaiser Permanente Southern California, the age-adjusted incidence rate for AFFs was 1.78/100,000 person-years with bisphosphonate exposure of 0.1 to 1.9 years and 113.1/100,000 person-years with bisphosphonate exposure of 8.0 to 9.9 years.^10^ A claims-based study using the Thomson Reuters MarketScan databases showed no significant associations between long-term use of risedronate or alendronate and the incidence of AFF.^11^ On the other hand, a recent study by Kaiser Permanente Southern California revealed an increase in the risk of AFF with longer duration of bisphosphonate use and a rapid decrease after bisphosphonate discontinuation.^12^ Risk factors for AFFs were investigated in 2,238 Japanese patients with hip and femoral shaft fractures; bisphosphonate use was shown to be a risk factor for developing AFF.^13^

The incidence of AFF is very low.^14^^,^^15^ Furthermore, comparative studies across antiresorptive drugs are necessary, as several studies reported that AFFs developed among patients treated with denosumab.^7^^,^^8^ To our knowledge, incidence rates of AFFs among users of antiresorptive drugs other than bisphosphonates and denosumab have not been reported. Therefore, a population-based, large-scale database study is an appealing approach to explore correlation between antiresorptive drug use and AFF incidence. The purpose of this study was to investigate the precise incidence rate of AFF in a nation-wide population using the National Database of Health Insurance Claims and Specific Health Checkups of Japan (NDB)^16^ and the relationship between AFF incidence and the use of antiresorptive drugs, including bisphosphonates, for the treatment of osteoporosis, which was cited as a risk factor for AFF. In addition, all femoral fractures were investigated in the same cohort to reveal the effectiveness of each antiresorptive drug from a risk–benefit perspective.

## METHODS

### Study design and data source

This was a cohort study using data from the NDB, a claims database maintained by the Japanese Ministry of Health, Labour and Welfare (MHLW) based on the Elderly Health Care Security Act.^16^ The NDB is a database of monthly claims for health insurance in Japan that started in April 2009. It includes all procedural codes, International Statistical Classification of Diseases, 10^th^ Revision (ICD-10) codes, and prescriptions from inpatient and outpatient services. Data on regular health examinations have been collected since April 2008. The database covers more than 95% claims issued from insured citizens in Japan (approximately 130 million people), except for cases covered by workers’ accident compensation insurance, automobile accident insurance, public assistance, or incurring private expenses.

In this study, we obtained a dataset with personal identification numbers, sex, age-group codes, records of prescriptions, records of procedures, diagnostic codes, and outcome codes (cured, died, transferred, or other) from the NDB between April 2009 and October 2016. The NDB provides two personal identification numbers (ID1, generated from the insurance identification number, birth date, and sex; ID2, generated from name, birth date, and sex) and the definition of the identifier used for linkage of records in this study is available in eMaterial 1. A list of ICD-10 codes used for the identification of fractures, osteoporosis, and comorbidities is available in eMaterial 2.

### Study population

The study population consisted of patients with new use of bisphosphonates, selective estrogen receptor modulators (SERMs), estrogens, calcitonins, and denosumab without a prior femoral fracture. Index month was defined for each patient as the month of the first prescription of any antiresorptive drug from April 2010 through August 2016, which means patients were followed for at most 6 years. Patients with new use were defined as those who had ICD-10 codes for osteoporosis (M81) in any record, records of prescription for antiresorptive drugs, and a period free of prescriptions for antiresorptive drugs of at least 3 months before the index month. Patients who had ICD-10 codes for any femoral fracture prior to 2 months after the index month were excluded. Codes assigned for suspected disease were not used for the identification of patients.

### Exposure

Drug exposure status was determined according to the records of prescriptions on a person-year basis. Person-months were classified into bisphosphonate monotherapy (alendronate, risedronate, minodronate, ibandronate, or etidronate), SERM monotherapy (raloxifene or bazedoxifene), estrogen monotherapy (estradiol, estriel, or conjugated estrogen), calcitonin monotherapy (elcatonin or salmon calcitonin), denosumab monotherapy, or combination therapy. For example, if a patient used a bisphosphonate drug from the first to the third month and also used a SERM drug only for the second month, the first, second, and third months were classified as bisphosphonate monotherapy, bisphosphonate and SERM combination therapy, and bisphosphonate monotherapy, respectively.

### Comorbidities

We identified comorbidities in each patient according to the Charlson Comorbidity Index^17^ and Elixhauser Comorbidity Index^18^ based on ICD-10 codes. Patients were considered to have a comorbidity if they had an ICD-10 code for the comorbidity during the 12 months before the index month. Only results using the Charlson Comorbidity Index were reported because results from the Elixhauser Comorbidity Index were similar.

### Outcome and validation study

The primary outcome was incidence of (non-traumatic) femoral shaft fracture. In our validation study,^19^ the definition of the primary outcome was intended to exclude traumatic fractures. However, it is impossible to identify all non-traumatic fractures from claims data, so we chose to use an outcome definition for femoral shaft fracture as the proxy for non-traumatic femoral shaft fracture. The secondary outcome was the incidence of (non-traumatic) all femoral fracture. Femoral shaft fractures were identified based on ICD-10 codes S72.3, S72.4 (supracondylar femur fracture), and S72.9 (femoral failure fracture). The ICD-10 codes for all femoral fractures are provided in eMaterial 2.

Femoral fractures were initially identified based on the ICD-10 codes in the claims data and a specialist in orthopedics (HH) reviewed the disease names of potential fracture cases. Codes assigned for suspected disease were not used. Definitions of femoral shaft fractures based on claims data in Japan have not been previously validated. Therefore, we performed a separate validation study to assess the relationship between fractures based on claims data and fractures based on electronic medical records and confirmed that sensitivity and specificity for femoral shaft fracture were 82.1% and 100.0%, respectively.^19^ The definition of femoral shaft fracture in this study is different from publication^19^ in that S72.9 (femoral failure fracture) was added in this study based on clinical consideration but the impact of modification in the analysis is small because femoral failure fracture is rare. The influence of traumatic fractures on study results would be small because incidence rate of traumatic fracture was low in patients older than 50 years, and sensitivity and specificity of identifying non-traumatic femoral shaft fractures based on claims data exceed 90%.^19^

The number of person-years used to calculate incidence was based on the month of the first femoral fracture after the index month, month of the last claims record, or month of death. Follow-up of fracture was terminated when the first fracture event occurred. To avoid reverse causation, the fracture events do not include fractures recorded in the index month and the next month.

### Statistical analysis

The background characteristics of patients were expressed as numbers and percentages for categorical variables, or as means and standard deviation for continuous variables. Crude incidence rates per 100,000 person-years and 95% confidence intervals (CIs) were calculated using the person-year method. Differences in incidence rates across antiresorptive drugs were expressed as adjusted incidence rate ratios with 95% CIs estimated using Poisson regression, with adjustment for sex, age, and Charlson Comorbidity Index. The same analyses were performed through categorizing the antiresorptive drugs according to their ingredients to explore the heterogeneous effects among ingredients. Furthermore, for bisphosphonates, an analysis where patients taking bisphosphonates were categorized by prescription period was also performed to explore the heterogeneous effects among the periods of bisphosphonates use. All analyses using SAS software version 9.4 (SAS Institute, Cary, NC, USA).

### Ethics statement

Because the data were completely de-identified before being provided to researchers, this study was exempt from obtaining individual informed consent according to the Ethical Guidelines on Biomedical Research Involving Human Subjects by the Japanese Ministry of Education, Culture, Sports, Science and Technology and the MHLW. The study protocol was approved by the ethics committee of the Public Health Research Foundation and the National Center for Geriatrics and Gerontology Ethics Committee (approval no. 971).

## RESULTS

**Figure 1.**
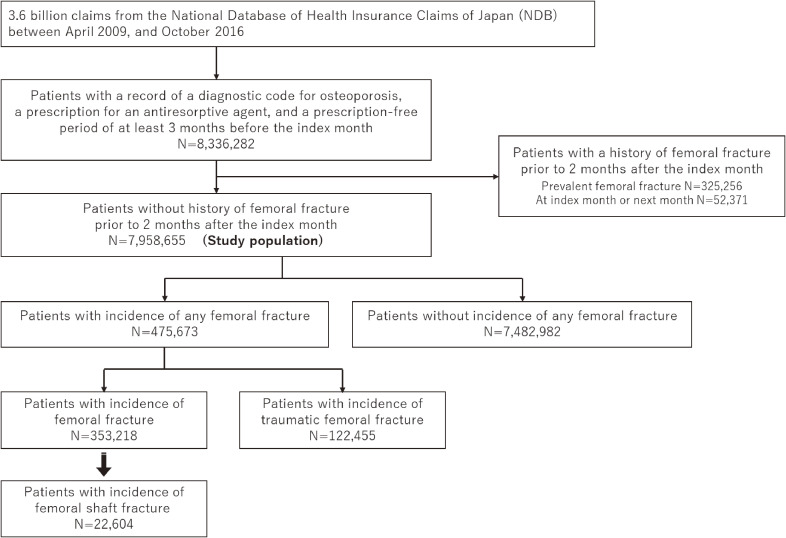
Flowchart showing identification of patients treated with antiresorptive drugs and incidence of femoral shaft fracture

Between 2009 and 2016, 8,336,282 patients with new use of antiresorptive drugs in Japan with available claims data were identified. After excluding patients with any femoral fracture prior to 2 months after the index month, the cohort consisted of 7,958,655 patients (Figure 1). The baseline characteristics of the 7,958,655 patients during the index month are shown in Table 1. In the cohort, 88.4% were women and 51.2% were aged 75 years or older. Common comorbidities included diabetes (50.2%); prior non-metastatic malignancy, leukemia, or lymphoma (34.0%); peptic ulcer disease (28.1%); cerebrovascular disease (27.8%); chronic pulmonary disease (25.0%); and congestive heart failure (21.1%).

**Table 1.** Background characteristics of patients treated with antiresorptive drugs (*n* = 7,958,655)

	Number	%
Sex		
Man	919,852	11.6
Woman	7,038,803	88.4
Age, years		
≤49	248,646	3.1
50–64	1,191,336	15.0
65–74	2,443,428	30.7
≥75	4,075,245	51.2
Charlson Comorbidity Index (mean and SD)	Mean: 2.41	SD: 2.68
Myocardial infarction	384,057	4.8
Congestive heart failure	1,680,252	21.1
Peripheral vascular disease	1,446,858	18.2
Cerebrovascular disease	2,213,554	27.8
Dementia	450,330	5.7
Chronic pulmonary disease	1,986,220	25.0
Rheumatologic disease	1,162,932	14.6
Peptic ulcer disease	2,234,731	28.1
Mild liver disease	1,377,080	17.3
Diabetes without chronic complications	3,531,233	44.4
Diabetes with chronic complications	464,211	5.8
Hemiplegia or paraplegia	78,749	1.0
Renal disease	334,630	4.2
Any malignancy, including leukemia and lymphoma	2,708,376	34.0
Moderate or severe liver disease	57,466	0.7
Metastatic solid tumor	387,523	4.9
AIDS/HIV	27,450	0.3

AIDS/HIV, acquired immunodeficiency syndrome/human immunodeficiency virus; SD, standard deviation.

When patients were classified according to the drug with the longest prescription period, the proportions of patients by drug type were 60.7% for bisphosphonate monotherapy, 16.2% for SERM monotherapy, 2.8% for estrogen monotherapy, 11.5% for calcitonin monotherapy, 2.6% for denosumab monotherapy, and 6.3% for combination therapy. Among patients on bisphosphonate monotherapy or SERM monotherapy, the prescription period was 2 years or more for 42.4% and 45.4% of patients, respectively. Transient use (ie, less than 1 year) was common among patients on estrogen monotherapy (69.4%) and calcitonin monotherapy (76.9%).

Femoral shaft fractures and all femoral fractures were identified in 22,604 and 353,218 patients, corresponding to incidence of 70.7/100,000 and 1,129.8/100,000 person-years, respectively. Femoral shaft fractures accounted for 6.4% of all femoral fractures. Table 2 summarizes age-specific and sex-specific incidence rates of femoral shaft and all femoral fractures. Age-specific incidence rates of femoral shaft fracture was 30.1/100,000 person-years for patients younger than 65 years, 47.7/100,000 person-years for patients aged 65–74 years, and 99.0/100,000 person-years for patients aged 75 years or older. Sex-specific incidence of femoral shaft fracture per 100,000 person-years was 74.8 for women and 30.1 for men. The sex difference in the frequency of femoral shaft fractures (incidence rate ratio 2.48; 95% CI, 2.32–2.66) was much larger than the sex difference for typical fractures (incidence rate ratio 1.17; 95% CI, 1.16–1.19).

**Table 2.** Incidence rates of femoral shaft fractures and all femoral fractures in patients treated with antiresorptive drugs (*n* = 7,958,655)

	Incidence	PYs	Incidence rate per 100,000 PYs (95% CI)		Adjusted incidence rate ratio^*^ (95% CI)	
Non-traumatic femoral shaft fractures	22,604	31,967,234	70.7	(69.8–71.6)		
Sex						
Man	873	2,899,938	30.1	(28.2–32.2)	(ref.)	
Woman	21,731	29,067,296	74.8	(73.8–75.8)	2.48	(2.32–2.66)
Age, years						
≤49	258	901,664	28.6	(25.3–32.3)	(ref.)	
50–64	1,440	4,735,766	30.4	(28.9–32)	1.06	(0.93–1.21)
65–74	4,799	10,063,681	47.7	(46.4–49.1)	1.67	(1.47–1.89)
≥75	16,107	16,266,123	99.0	(97.5–100.6)	3.46	(3.06–3.91)
Charlson Comorbidity Index						
Above mean	8,669	10,816,782	80.1	(78.5–81.8)	1.22	(1.18–1.25)
At or below mean	13,935	21,150,452	65.9	(64.8–67)	(ref.)	

Non-traumatic all femoral fractures	353,218	31,264,530	1,129.8	(1,126.1–1,133.5)		
Sex						
Man	27,338	2,855,601	957.3	(946.1–968.8)	(ref.)	
Woman	325,880	28,408,929	1,147.1	(1,143.2–1,151.1)	1.20	(1.18–1.21)
Age, years						
≤49	1,883	897,897	209.7	(200.5–219.4)	(ref.)	
50–64	14,501	4,706,332	308.1	(303.1–313.2)	1.47	(1.4–1.54)
65–74	57,271	9,947,613	575.7	(571–580.5)	2.75	(2.62–2.87)
≥75	279,563	15,712,688.26	1,779.2	(1,772.6–1,785.8)	8.48	(8.11–8.88)
Charlson Comorbidity Index						
Above mean	152,743	10,520,472.13	1,451.9	(1,444.6–1,459.2)	1.50	(1.49–1.51)
At or below mean	200,475	20,744,057.93	966.4	(962.2–970.7)	(ref.)	

CI, confidence interval; PY, person-year.

^*^Adjusted for age, sex, and Charlson Comorbidity Index.

Table 3 shows incidence rates and incidence rate ratios for femoral shaft fractures in patients taking versus not taking antiresorptive drugs. The incidence of femoral shaft fracture was 70.7/100,000 person-years for patients taking bisphosphonates, 36.0/100,000 person-years for patients taking SERMs, 11.5/100,000 person-years for patients taking estrogens, 63.8/100,000 person-years for patients taking calcitonins, and 70.6/100,000 person-years for patients taking denosumab. Somewhat unexpectedly, most antiresorptive drugs were not associated with a higher risk of femoral shaft fracture after adjusting for sex, age, and Charlson Comorbidity Index. Adjusted incidence rate ratios for patients taking versus not taking each type of antiresorptive drug were 1.00 (95% CI, 0.98–1.03) for bisphosphonates, 0.46 (95% CI, 0.44–0.48) for SERMs, 0.24 (95% CI, 0.18–0.32) for estrogens, 0.75 (95% CI, 0.71–0.79) for calcitonins, and 0.93 (95% CI, 0.84–1.03) for denosumab. In an analysis where patients taking antiresorptive drugs were categorized according to ingredients, only alendronate was significantly associated with an increased risk of femoral shaft fracture (adjusted incidence rate ratio 1.18; 95% CI, 1.14–1.22). Furthermore, in an analysis where patients taking bisphosphonates were categorized by prescription period, the adjusted incidence rate ratios were 1.09 (95% CI, 1.06–1.12) for patients treated for less than 1 year, 1.35 (95% CI, 1.32–1.39) for patients treated for 1–2 years, and 0.67 (95% CI, 0.65–0.70) for patients treated for 2 years or longer.

**Table 3.** Incidence rates of femoral shaft fractures according to antiresorptive drugs

	Incidence	PYs	Incidence rate per 100,000 PYs (95% CI)		Adjusted incidence rate ratio^*^ (95% CI)	
Treated with bisphosphonates	9,953	14,082,533	70.7	(69.3–72.1)	1.00	(0.98–1.03)
Not treated with bisphosphonates	12,639	17,675,648	71.5	(70.3–72.8)	(ref.)	
Alendronate	5,132	6,262,454	81.9	(79.7–84.2)	1.18	(1.14–1.22)
Risedronate	2,801	4,544,522	61.6	(59.4–64)	0.87	(0.83–0.9)
Minodronate	1,698	2,857,845	75.9	(56.7–62.3)	0.85	(0.81–0.89)
Ibandronate	382	503,152	59.4	(68.7–83.9)	0.95	(0.86–1.06)
Etidronate	16	20,537	77.9	(47.7–127.2)	1.14	(0.7–1.86)

Treated with SERMs	1,499	4,162,308	36.0	(34.2–37.9)	0.46	(0.44–0.48)
Not treated with SERMs	21,093	27,595,873	76.4	(75.4–77.5)	(ref.)	
Raloxifene	1,137	3,052,672	37.2	(35.1–39.5)	0.47	(0.44–0.5)
Bazedoxifene	370	1,123,611	32.9	(29.7–36.5)	0.45	(0.4–0.49)

Treated with estrogens	49	424,989	11.5	(8.7–15.3)	0.24	(0.18–0.32)
Not treated with estrogens	22,543	31,333,192	71.9	(71–72.9)	(ref.)	
Estradiol	35	163,952	21.3	(15.3–29.7)	0.36	(0.26–0.5)

Treated with calcitonins	1,557	2,440,974	63.8	(60.7–67)	0.75	(0.71–0.79)
Not treated with calcitonins	21,035	29,317,207	71.7	(70.8–72.7)	(ref.)	
Elcatonin	1,538	2,405,308	63.9	(60.8–67.2)	0.75	(0.71–0.79)
Salmon calcitonin	20	37,158	53.8	(34.7–83.4)	0.62	(0.4–0.96)

Treated with denosumab	350	495,895	70.6	(63.6–78.4)	0.93	(0.84–1.03)
Not treated with denosumab	22,242	31,262,286	71.1	(70.2–72.1)	(ref.)	

CI, confidence interval; PY, person-year; SERM, selective estrogen receptor modulator.

^*^Adjusted for age, sex, and Charlson Comorbidity Index.

The incidence rates of all femoral fracture according to antiresorptive drugs are shown in Table 4. Significant reductions in fracture risk were observed for antiresorptive drugs, including alendronate.

**Table 4.** Incidence rates of all femoral fractures according to antiresorptive drugs

	Incidence	PYs	Incidence rate per 100,000 PYs (95% CI)		Adjusted incidence rate ratio^*^ (95% CI)	
Treated with bisphosphonates	121,420	13,860,964	876.0	(871.1–880.9)	0.67	(0.66–0.67)
Not treated with bisphosphonates	231,583	17,207,562	1,345.8	(1,340.4–1,351.3)	(ref.)	
Alendronate	55,602	6,166,595	901.7	(894.2–909.2)	0.70	(0.69–0.71)
Risedronate	38,008	4,479,583	848.5	(840.0–857.0)	0.65	(0.64–0.65)
Minodronate	22,899	2,809,122	1,181.7	(804.7–825.8)	0.63	(0.63–0.64)
Ibandronate	5,771	488,362	815.2	(1,151.6–1,212.6)	0.77	(0.75–0.79)
Etidronate	139	20,291	685.0	(580.1–808.9)	0.54	(0.46–0.64)

Treated with SERMs	32,922	4,102,689	802.4	(793.8–811.2)	0.70	(0.70–0.71)
Not treated with SERMs	320,081	26,965,837	1,187.0	(1,182.9–1,191.1)	(ref.)	
Raloxifene	25,511	3,008,851	847.9	(837.5–858.3)	0.73	(0.72–0.74)
Bazedoxifene	7,547	1,107,476	681.5	(666.3–697.0)	0.64	(0.63–0.66)

Treated with estrogens	1,060	422,825	250.7	(236.0–266.3)	0.44	(0.41–0.46)
Not treated with estrogens	351,943	30,645,701	1,148.4	(1,144.6–1,152.2)	(ref.)	
Estradiol	691	162,449	425.4	(394.8–458.3)	0.50	(0.46–0.53)

Treated with calcitonins	29,793	2,401,264	1,240.7	(1,226.7–1,254.9)	0.87	(0.86–0.88)
Not treated with calcitonins	323,210	28,667,262	1,127.5	(1,123.6–1,131.3)	(ref.)	
Elcatonin	29,374	2,366,068	1,241.5	(1,227.4–1,255.7)	0.87	(0.86–0.88)
Salmon calcitonin	439	36,665	1,197.3	(1,090.4–1,314.7)	0.82	(0.75–0.90)

Treated with denosumab	4,915	475,825	1,032.9	(1,004.5–1,062.2)	0.83	(0.80–0.85)
Not treated with denosumab	348,088	30,592,701	1,137.8	(1,134.0–1,141.6)	(ref.)	

CI, confidence interval; PY, person-year; SERM, selective estrogen receptor modulator.

^*^Adjusted for age, sex, and Charlson Comorbidity Index.

## DISCUSSION

This is the first report indicating the nationwide incidence of femoral shaft fracture and total femoral fractures in patients who treated with antiresorptive drugs in Japan. The estimated incidence rates for femoral shaft and all femoral fractures were higher than the incidence rates reported in an Asian population.^13^ Our study revealed that over half of the patients with osteoporosis using antiresorptive drugs in Japan were aged 75 years or older, and consequently, the mean age of the study population was higher than in previous studies.^10^^–^^13^ Furthermore, as also reported in the previous study,^12^ increasing age is associated with an increased risk for both femoral shaft and all femoral fractures. These results could account for the relatively higher incidence rates for femoral shaft and all femoral fractures.

In this study, most antiresorptive drugs were not associated with an increased risk of femoral shaft fracture during follow-up, which was at most 6 years. Significant increases in the risk of femoral shaft fracture were observed in patients treated with alendronate, but not in patients treated with other antiresorptive drugs, including denosumab. Our findings are consistent with a previous review that indicated a higher risk of AFF with alendronate than risedronate.^20^ In general, antiresorptive drugs decrease the risk of fractures by inhibiting osteoclasts. However, in the case of bisphosphonate, long-term accumulation to bone is thought to increase bone fragility. The increased risk associated with alendronate might be explained by its higher affinity for bone minerals than the other bisphosphonates.^21^

Increasing age, Asian race, and duration of bisphosphonate use are known as risk factors for femoral shaft fracture among patients taking bisphosphonates.^12^ In this study, no clear relationship between longer duration of bisphosphonate therapy and increased risk of femoral shaft fracture was observed during the relatively short follow-up. Management of treatment duration might contribute to the incidence of femoral shaft fracture.

One strength of this study was that the risk of femoral shaft fracture and all femoral fractures was assessed for a wide range of antiresorptive drugs in detail. The results suggested that there can be heterogeneity in the risk of femoral shaft fracture within the class of bisphosphonates. While alendronate was used in more than half or the vast majority of bisphosphonate exposure in previous studies,^11^^,^^12^ alendronate accounted for less than half of the bisphosphonate exposure in this study based on person-years of exposure. This suggested that further research on associations between each specific antiresorptive drug and femoral shaft fractures is needed.

One study limitation is that it was not possible to exclude potential bias due to confounding and informative censoring. Specifically, among the known risk factors of atypical femur fracture, data on long-term use of glucocorticoids, height, and weight were not available, so they were not adjusted for in our analysis. Furthermore, the duration of follow-up was shorter than in previous studies. The lack of positive correlations between the incidence of femoral shaft fracture and treatment duration in our study might be attributable to these differences in follow-up. Another limitation is the diagnostic accuracy of fracture based on ICD-10 codes without radiographs. For example, fractures recorded in the index months could actually occur before initiation of pharmacological treatment. The definitions of the outcomes do not include fractures recorded in the index month and the next month to avoid reverse causation and we confirmed other definitions lead to similar results (data not shown). Finally, we estimated an adjusted incidence rate ratio for each antiresorptive drug but the choice of non-users of a specific drug as the referent group may be criticized. Specifically, it is possible that non-users of the non-bisphosphonate medications switched to bisphosphonates and some person-years were actually exposed to bisphosphonates, yielding a potential of bias toward null. Furthermore, the dose-response relationships between prescription period of bisphosphonates and femoral shaft fracture in our study were not monotonic, unlike previous studies. This may be attributable to shorter use of bisphosphonates compared with other studies.^10^^–^^12^ More than half of patients in this study were exposed to bisphosphonates less than 2 years. Thus, it is not reasonable to rule out the effects of bisphosphonates on the risk of femoral shaft fracture based on our findings. In conclusion, the incidence rates of femoral shaft fracture varied across patients treated with different antiresorptive drugs. Further research on a specific antiresorptive drug can increase understanding of the risk of femoral shaft fracture.

## SUPPLEMENTARY MATERIAL

eMaterial 1. Definition of the identifier used for linkage of records

eMaterial 2. List of ICD-10 codes used for identification of fractures, osteoporosis, comorbidities, and prescription of antiresorptive drugs
