# Supplementary material for: Antiresorptive Drugs and the Risk of Femoral Shaft Fracture in Men and Women With Osteoporosis: A Cohort Study Using the National Database of Health Insurance Claims of Japan
Source: J Epidemiol. 2023 Dec 5;33(12):633–9. doi: 10.2188/jea.JE20220099 (PMC10635809; doi:10.2188/jea.JE20220099)
Supplement: Supplementary file 1 [file je-33-633-s001.pdf]

eMaterial 1. Definition of the identifier used for linkage of records

The NDB provides two personal identification numbers. ID1 is generated from the insurance identification number, birth date, and sex. ID2 is generated from name, birth date, and sex. ID1 can change when beneficiaries change their jobs and their insurers change, and ID2 can change when beneficiaries’ names changed after life events. Furthermore, both ID1 and ID2 are prone to change due to variations in spelling or formatting of names. Thus, an individual can have various ID1s and ID2s. In this study, identifier used for linkage of records in NDB was defined by the following algorithm utilizing possible combination patterns of ID1 and ID2.

1. Extract ID1s that were connected to a single ID2.
2. Extract ID2s that were connected to any of ID1s extracted in the previous step.
3. Extract ID1s that were connected to any of ID2s extracted in the previous step.
4. Repeat the step 2 and 3 until no additional IDs were identified.
5. Assign new identifier to records with ID1 and ID2 identified in the previous step.

ID2	ID1
333333	12

ID1	ID2
12	333333
12	444444

ID2	ID1
333333	12
444444	35
444444	12
444444	86
444444	23
444444	260
444444	281

ID1	ID2
12	333333
35	444444
12	444444
86	444444
23	444444
23	666666
260	444444
281	444444
281	666666

ID2	ID1
333333	12
666666	155
666666	23
666666	281
444444	35
444444	12
444444	86
444444	23
444444	260
444444	281

ID1	ID2
12	333333
155	666666
23	666666
281	666666
35	444444
12	444444
86	444444
23	444444
260	444444
281	444444

eMaterial 2. List of ICD-10 codes used for identification of fractures, osteoporosis, comorbidities, and prescription of antiresorptive drugs

Variable	Code
Femoral shaft fracture*	S72.3, S72.4 (supracondylar femur fracture), S72.9 (femoral failure fracture)
Total femoral fracture*	S72.0, S72.1, S72.2, S72.3, S72.4, S72.9
Osteoporosis	M81
Myocardial infarction	I210, I211, I212, I213, I214, I219, I220, I221, I228, I229, I252
Congestive heart failure	I099, I110, I255, I420, I425, I426, I427, I428, I429, I500, I501, I509, P290
Peripheral vascular disease	I700, I701, I702, I708, I709, I710, I711, I712, I713, I714, I715, I716, I718, I719, I731, I738, I739, I771, K551, K558, K559, Z958
Cerebrovascular disease	G450, G451, G453, G454, G458, G459, H340, I600, I601, I602, I603, I604, I605, I606, I607, I608, I609, I610, I611, I613, I614, I615, I616, I618, I619, I620, I621, I629, I630, I631, I632, I633, I634, I635, I636, I638, I639, I64, I650, I651, I652, I653, I658, I660, I661, I662, I663, I668, I669, I670, I671, I672, I673, I674, I675, I676, I677, I678, I679, I690, I691, I693, I694
Dementia	F010, F011, F012, F019, F03, F051, G300, G301, G308, G309, G311
Chronic pulmonary disease	I279, J40, J410, J411, J42, J430, J431, J432, J439, J448, J449, J450, J451, J458, J459, J46, J47, J60, J61, J628, J630, J631, J632, J633, J634, J635, J64, J65, J660, J661, J670, J671, J672, J673, J674, J675, J676, J677, J678, J679, J684, J701, J703
Rheumatic disease	M0500, M0510, M0520, M053, M0530, M0590, M0600, M0610, M0620, M0630, M0640, M0680, M0684, M0690, M0691, M0692, M0693, M0694, M0695, M0696, M0697, M0698, M320, M321, M329, M330, M331, M332, M339, M340, M341, M348, M349, M351, M353
Peptic ulcer disease	K250, K251, K252, K253, K254, K255, K256, K257, K259, K260, K261, K262, K263, K264, K265, K266, K267, K269, K270, K277, K279, K284, K285, K287, K289
Mild liver disease	B181, B182, B189, K700, K701, K702, K703, K709, K713, K730, K732, K738, K739, K740, K741, K743, K744, K745, K746, K760, K762, K763, K764, K768
Diabetes without chronic complication	E10, E100, E101, E102, E103, E104, E105, E106, E107, E109, E11, E110, E111, E112, E113, E114, E115, E116, E119, E12, E13, E130, E131, E132, E133, E134, E135, E136, E137, E139, E14, E140, E141, E142, E143, E144, E145, E146, E149
Diabetes with chronic complication	E102 E103 E104 E105 E107 E112 E113 E114 E115 E117 E132 E133 E134 E135 E137 E142 E143 E144 E145

Hemiplegia or paraplegia	G114 G801 G802 G810 G811 G819 G820 G821 G822 G823 G824 G825 G830 G831 G832 G833 G834 G839
Renal disease	I120, N032, N033, N034, N036, N037, N052, N053, N054, N055, N056, N057, N180, N188, N189, N19, N250, Z940
Any malignancy, including lymphoma and leukemia, except for malignant neoplasm of skin	C000, C001, C002, C003, C004, C006, C008, C009, C01, C020, C021, C022, C029, C030, C031, C039, C040, C041, C049, C050, C051, C052, C059, C060, C061, C062, C069, C07, C080, C081, C089, C090, C091, C099, C100, C101, C102, C103, C104, C109, C110, C111, C112, C113, C119, C12, C130, C131, C132, C139, C140, C150, C151, C152, C153, C154, C155, C158, C159, C160, C161, C162, C163, C164, C165, C166, C169, C170, C171, C172, C179, C180, C181, C182, C183, C184, C185, C186, C187, C189, C19, C20, C210, C211, C220, C221, C222, C223, C224, C227, C229, C23, C240, C241, C249, C250, C251, C252, C253, C254, C257, C258, C259, C261, C269, C300, C301, C310, C311, C312, C313, C319, C320, C321, C322, C323, C329, C33, C340, C341, C342, C343, C348, C349, C37, C380, C381, C382, C383, C384, C400, C401, C402, C403, C410, C411, C412, C413, C414, C419, C430, C431, C432, C433, C434, C435, C436, C437, C438, C439, C450, C451, C452, C459, C469, C470, C471, C472, C473, C474, C475, C476, C479, C480, C481, C482, C490, C491, C492, C493, C494, C495, C496, C499, C50, C500, C501, C502, C503, C504, C505, C506, C508, C509, C510, C511, C512, C519, C52, C538, C539, C540, C541, C542, C543, C549, C55, C56, C570, C579, C58, C600, C601, C602, C609, C61, C620, C621, C629, C630, C631, C632, C637, C639, C64, C65, C66, C670, C671, C672, C673, C674, C675, C676, C677, C679, C680, C681, C690, C691, C692, C693, C694, C696, C700, C701, C709, C710, C711, C712, C713, C714, C715, C716, C717, C719, C720, C721, C723, C724, C725, C729, C73, C740, C741, C749, C750, C751, C752, C753, C754, C755, C760, C761, C762, C763, C764, C765, C810, C811, C812, C813, C817, C819, C820, C821, C822, C827, C829, C830, C831, C832, C833, C834, C835, C836, C837, C838, C839, C840, C841, C842, C843, C844, C845, C851, C857, C859, C880, C881, C882, C883, C887, C900, C901, C902, C910, C911, C913, C914, C915, C917, C919, C920, C921, C923, C924, C925, C927, C929, C930, C931, C939, C940, C942, C943, C945, C947, C950, C951, C957, C959, C960, C961, C962, C967, C97
Moderate or severe liver disease	I850, I859, I864, K704, K711, K721, K729, K765, K766, K767
Metastatic solid tumor	C770, C771, C772, C773, C774, C775, C778, C779, C780, C781, C782, C783, C784, C785, C786,

	C787, C788, C790, C791, C792, C793, C794, C795, C796, C797, C798, C80
AIDS/HIV	B200, B202, B204, B206, B210, B211, B212, B220, B221, B222, B24

AIDS/HIV, acquired immunodeficiency syndrome/human immunodeficiency virus; ICD-10, International Classification of Diseases, 10th revision.

*Traumatic femoral fractures were identified based on the detailed disease names in the claims data and were not included in the definition of the outcome.
